# Retraction Note: Amelioration of obsessive-compulsive disorder in three mouse models treated with one epigenetic drug: unraveling the underlying mechanism

**DOI:** 10.1038/s41598-021-84474-5

**Published:** 2021-02-23

**Authors:** German Todorov, Karthikeyan Mayilvahanan, David Ashurov, Catarina Cunha

**Affiliations:** 1 Emotional Brain Institute, Nathan Kline Institute, Orangeburg, NY USA; 2 Department of Neurobiology and Behavior, Stony Brook University School of Medicine, Stony Brook, NY USA

Retraction of: *Scientific Reports* 10.1038/s41598-019-45325-6, publisher online 19 June 2019

The Editors have retracted this Article.

An investigation by the Research Integrity Office at Stony Brook University has concluded that:

- some of the methodological and experimental descriptions in the paper show significant overlap with NIH grant application R01-NS104089, NARSAD grant application 23840, and a previously published article by Plotkin *et al*.^1^;
- the Authors incorrectly reported results for Slitrk5 knockout mice. Due to problems with genotyping of these animals, the genetic identity of the individual animals was impossible to determine;
- the Authors incorrectly reported results for Sapap3 knockout mice. The animals used in these experiments were Sapap3 conditional knock-in mice;
- the experimental conditions in the behavioural tests were also incorrectly reported: the experimenters were not blind to experimental conditions as reported in the paper.

The Editors therefore no longer have confidence in the integrity of the data presented in this Article.

Catarina Cunha disagrees with the retraction. German Todorov, Karthikeyan Mayilvahanan and David Ashurov did not respond to the correspondence about this retraction.
